# Diaqua­bis­[1-ethyl-6-fluoro-4-oxo-7-(piperazin-1-yl)-1,4-dihydroquinoline-3-carboxyl­ato]magnesium(II) hexa­hydrate

**DOI:** 10.1107/S1600536810039437

**Published:** 2010-10-13

**Authors:** Ji-Feng Wen, Wen-Zhe Yin, Ya-Xian Qiao

**Affiliations:** a The Second Affiliated Hospital, Harbin Medical University, Harbin, 150086, People’s Republic of China; b Scientific and Technical Department, Harbin Medical University, Harbin, 150086, People’s Republic of China

## Abstract

In the title compound, [Mg(C_16_H_17_FN_3_O_3_)_2_(H_2_O)_2_]·6H_2_O, the Mg^2+^ ion (site symmetry 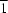) exhibits a distorted MgO_6_ octa­hedral geometry defined by two *O*,*O*-bidentate 1-ethyl-6-fluoro-1,4-dihydro-4-oxo-7-(1-piperazin­yl)-3-quinoline-carb­oxyl­ate (norf) anions and two water mol­ecules. In the crystal, O—H⋯O and O—H⋯N hydrogen bonds help to establish the packing.

## Related literature

For the cadmium, zinc and cobalt(II) complexes of the norf anion, see: Chen *et al.* (2001 ▶), Wang *et al.* (2004 ▶) and An *et al.* (2007 ▶), respectively. For background to the medicinal uses of Norfloxacin [H-norf or 1-ethyl-6-fluoro-1,4-dihydro-4-oxo-7-(1-piperazin­yl)-3-quinoline carb­oxy­lic acid], which is used to treat infections, see: Mizuki *et al.* (1996 ▶).
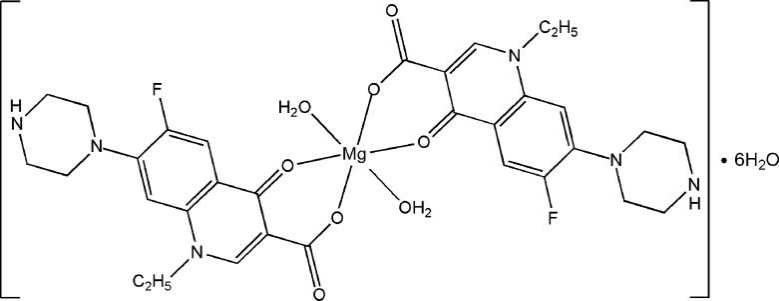


## Experimental

### 

#### Crystal data

[Mg(C_16_H_17_FN_3_O_3_)_2_(H_2_O)_2_]·6H_2_O
*M* *_r_* = 805.09
Triclinic, 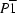

*a* = 5.0944 (10) Å
*b* = 13.785 (3) Å
*c* = 14.351 (3) Å
α = 112.06 (3)°
β = 97.59 (3)°
γ = 93.74 (3)°
*V* = 918.6 (3) Å^3^
*Z* = 1
Mo *K*α radiation
μ = 0.14 mm^−1^
*T* = 295 K
0.12 × 0.10 × 0.08 mm

#### Data collection

Bruker APEXII CCD diffractometer
Absorption correction: multi-scan (*SADABS*; Bruker, 2001 ▶) *T*_min_ = 0.984, *T*_max_ = 0.989
7196 measured reflections
3203 independent reflections
1774 reflections with *I* > 2σ(*I*)
*R*_int_ = 0.053

#### Refinement

*R*[*F* ^2^ > 2σ(*F* ^2^)] = 0.065
*wR*(*F* ^2^) = 0.249
*S* = 1.00
3203 reflections
278 parameters
14 restraints
H atoms treated by a mixture of independent and constrained refinement
Δρ_max_ = 0.38 e Å^−3^
Δρ_min_ = −0.41 e Å^−3^

### 

Data collection: *APEX2* (Bruker, 2004 ▶); cell refinement: *SAINT-Plus* (Bruker, 2001 ▶); data reduction: *SAINT-Plus*; program(s) used to solve structure: *SHELXS97* (Sheldrick, 2008 ▶); program(s) used to refine structure: *SHELXL97* (Sheldrick, 2008 ▶); molecular graphics: *SHELXTL* (Sheldrick, 2008 ▶); software used to prepare material for publication: *SHELXTL*.

## Supplementary Material

Crystal structure: contains datablocks I, global. DOI: 10.1107/S1600536810039437/hb5638sup1.cif

Structure factors: contains datablocks I. DOI: 10.1107/S1600536810039437/hb5638Isup2.hkl

Additional supplementary materials:  crystallographic information; 3D view; checkCIF report

## Figures and Tables

**Table 1:** Selected bond lengths (Å)

Mg1—O2	2.001 (3)
Mg1—O1	2.085 (3)
Mg1—O1*W*	2.094 (3)

**Table 2:** Hydrogen-bond geometry (Å, °)

*D*—H⋯*A*	*D*—H	H⋯*A*	*D*⋯*A*	*D*—H⋯*A*
N1—H1⋯O2*W*^i^	0.86	2.20	2.787 (7)	126
O1*W*—H1*W*⋯O2*W*	0.82 (1)	1.97 (1)	2.786 (6)	175 (3)
O1*W*—H2*W*⋯O1^ii^	0.82 (2)	2.09 (2)	2.901 (5)	175 (2)
O2*W*—H3*W*⋯O3^iii^	0.82 (5)	1.98 (5)	2.749 (6)	156 (4)
O2*W*—H4*W*⋯N1^iv^	0.82 (2)	2.36 (4)	3.121 (7)	154 (5)
O3*W*—H5*W*⋯O3^iii^	0.82 (4)	2.18 (4)	2.835 (6)	137 (5)
O3*W*—H6*W*⋯O4*W*	0.81 (15)	2.3 (2)	2.890 (8)	131 (19)
O4*W*—H8*W*⋯O3*W*^v^	0.82 (4)	2.31 (4)	2.888 (8)	128 (4)

Symmetry codes: (i) 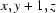; (ii) 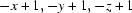; (iii) 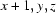; (iv) 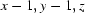; (v) 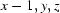.

**Fig. 1.**
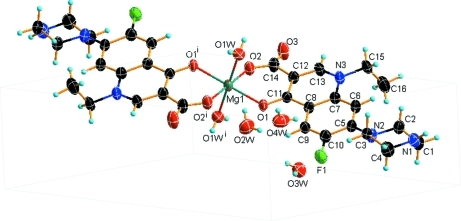
The molecular structure of (I), showing the Mg coordination with 50% displacement ellipsoids. Symmetry code: (i) -x, -y, 1 - z.

## supplementary crystallographic information

### Comment

Norfloxacin (*H*-norf, 1-ethyl-6-fluoro-1,4-dihydro-4-oxo-7-(1-piperazinyl) -3-quinoline carboxylic acid) is member of the class of quinolones that is used to treat infections (Mizuki *et al.*, 1996). Cadmium(II), zinc(II) and cobalt(II) derivatives of norf have been reported (Chen *et al.*, 2001; Wang *et al.*, 2004; An *et al.*,2007).

The title magnesium(II)-containing complex of norf, (I), is reported here.(Fig. 1).

The structure of (I) is built up from Mg^2+^ cations, norf ligands, coordinated water molecules, uncoordinated water molecules (Fig. 1). The manganese geometry is a slightly distorted octahedron.

The components of (I) are linked by O—H···O and O—H···N hydrogen bonds involving all the potential donors, generating a three-dimensional supramolecular network.

### Experimental

A mixture of Mg(NO_3_)_2_.2H_2_O (0.5 mmol), H-norf(0.6 mmol), and water (12 ml) was stirred for 30 min in air. The mixture was then transferred to a 25 ml Teflon reactor and kept at 433 K for 72 h under autogenous pressure. Upon cooling, colorless blocks of (I) were obtained from the reaction mixture.

### Refinement

The carbon-bound H atoms were positioned geometrically (C—H = 0.93–0.97 Å) and refined as riding with *U*_iso_(H) = 1.2*U*_eq_(C). The H atoms on the N and water molecules were located in a difference map and refined with a distance restraint of N—H = 0.90 (1) Å, O—H = 0.85 (1) Å,and the constraint *U*_iso_(H) = 1.5*U*_eq_(N,*O*).

### Crystal data

**Table:**

[Mg(C_16_H_17_FN_3_O_3_)_2_(H_2_O)_2_]·6H_2_O	*Z* = 1
*M**_r_* = 805.09	*F*(000) = 426
Triclinic, *P*1	*D*_x_ = 1.455 Mg m^−^^3^
Hall symbol: -P 1	Mo *K*α radiation, λ = 0.71073 Å
*a* = 5.0944 (10) Å	Cell parameters from 3203 reflections
*b* = 13.785 (3) Å	θ = 3.1–25.0°
*c* = 14.351 (3) Å	µ = 0.14 mm^−^^1^
α = 112.06 (3)°	*T* = 295 K
β = 97.59 (3)°	Block, colorless
γ = 93.74 (3)°	0.12 × 0.10 × 0.08 mm
*V* = 918.6 (3) Å^3^	

### Data collection

**Table:**

Bruker APEXII CCD diffractometer	3203 independent reflections
Radiation source: fine-focus sealed tube	1774 reflections with *I* > 2σ(*I*)
graphite	*R*_int_ = 0.053
phi and ω scans	θ_max_ = 25.0°, θ_min_ = 3.1°
Absorption correction: multi-scan (*SADABS*; Bruker, 2001)	*h* = −5→6
*T*_min_ = 0.984, *T*_max_ = 0.989	*k* = −16→15
7196 measured reflections	*l* = −17→17

### Refinement

**Table:**

Refinement on *F*^2^	Primary atom site location: structure-invariant direct methods
Least-squares matrix: full	Secondary atom site location: difference Fourier map
*R*[*F*^2^ > 2σ(*F*^2^)] = 0.065	Hydrogen site location: inferred from neighbouring sites
*wR*(*F*^2^) = 0.249	H atoms treated by a mixture of independent and constrained refinement
*S* = 1.00	*w* = 1/[σ^2^(*F*_o_^2^) + (0.130*P*)^2^ + 1.1685*P*] where *P* = (*F*_o_^2^ + 2*F*_c_^2^)/3
3203 reflections	(Δ/σ)_max_ < 0.001
278 parameters	Δρ_max_ = 0.38 e Å^−^^3^
14 restraints	Δρ_min_ = −0.41 e Å^−^^3^

### Special details

**Table:**

Geometry. All e.s.d.'s (except the e.s.d. in the dihedral angle between two l.s. planes) are estimated using the full covariance matrix. The cell e.s.d.'s are taken into account individually in the estimation of e.s.d.'s in distances, angles and torsion angles; correlations between e.s.d.'s in cell parameters are only used when they are defined by crystal symmetry. An approximate (isotropic) treatment of cell e.s.d.'s is used for estimating e.s.d.'s involving l.s. planes.
Refinement. Refinement of *F*^2^ against ALL reflections. The weighted *R*-factor *wR* and goodness of fit *S* are based on *F*^2^, conventional *R*-factors *R* are based on *F*, with *F* set to zero for negative *F*^2^. The threshold expression of *F*^2^ > σ(*F*^2^) is used only for calculating *R*-factors(gt) *etc*. and is not relevant to the choice of reflections for refinement. *R*-factors based on *F*^2^ are statistically about twice as large as those based on *F*, and *R*- factors based on ALL data will be even larger.

### Fractional atomic coordinates and isotropic or equivalent isotropic displacement parameters (Å^2^)

**Table:**

	*x*	*y*	*z*	*U*_iso_*/*U*_eq_	
C1	0.6643 (11)	1.0494 (5)	0.1860 (4)	0.0542 (15)	
H1A	0.8374	1.0250	0.1780	0.065*	
H1B	0.5932	1.0600	0.1249	0.065*	
C2	0.4804 (10)	0.9679 (4)	0.1990 (4)	0.0434 (12)	
H2A	0.3051	0.9910	0.2043	0.052*	
H2B	0.4622	0.9021	0.1399	0.052*	
C3	0.6286 (11)	1.0513 (4)	0.3807 (4)	0.0485 (13)	
H3A	0.7095	1.0396	0.4401	0.058*	
H3B	0.4582	1.0765	0.3932	0.058*	
C4	0.8076 (12)	1.1339 (4)	0.3651 (5)	0.0563 (15)	
H4A	0.8267	1.2000	0.4239	0.068*	
H4B	0.9833	1.1117	0.3584	0.068*	
C5	0.4638 (9)	0.8637 (3)	0.3026 (3)	0.0347 (11)	
C6	0.2314 (9)	0.8028 (4)	0.2407 (4)	0.0379 (11)	
H6	0.1484	0.8213	0.1891	0.046*	
C7	0.1195 (9)	0.7150 (3)	0.2534 (3)	0.0336 (11)	
C8	0.2283 (9)	0.6871 (3)	0.3335 (3)	0.0333 (11)	
C9	0.4682 (9)	0.7473 (4)	0.3948 (3)	0.0360 (11)	
H9	0.5519	0.7296	0.4469	0.043*	
C10	0.5770 (9)	0.8300 (4)	0.3787 (4)	0.0385 (11)	
C11	0.1060 (8)	0.6013 (3)	0.3537 (3)	0.0324 (10)	
C12	−0.1318 (9)	0.5427 (4)	0.2836 (3)	0.0370 (11)	
C13	−0.2237 (10)	0.5729 (4)	0.2062 (4)	0.0390 (12)	
H13	−0.3732	0.5321	0.1601	0.047*	
C14	−0.2863 (10)	0.4475 (4)	0.2873 (4)	0.0413 (12)	
C15	−0.2428 (10)	0.6777 (4)	0.1028 (4)	0.0435 (12)	
H15A	−0.2341	0.7535	0.1229	0.052*	
H15B	−0.4297	0.6488	0.0853	0.052*	
C16	−0.1147 (13)	0.6332 (5)	0.0103 (4)	0.0629 (16)	
H16A	0.0675	0.6649	0.0255	0.094*	
H16B	−0.2094	0.6480	−0.0444	0.094*	
H16C	−0.1201	0.5583	−0.0097	0.094*	
F1	0.8150 (5)	0.8827 (2)	0.4368 (2)	0.0517 (8)	
Mg1	0.0000	0.5000	0.5000	0.0334 (6)	
N1	0.6945 (10)	1.1490 (3)	0.2742 (4)	0.0570 (13)	
H1	0.6518	1.2077	0.2725	0.068*	
N2	0.5853 (8)	0.9511 (3)	0.2908 (3)	0.0410 (10)	
N3	−0.1186 (8)	0.6557 (3)	0.1905 (3)	0.0392 (10)	
O1	0.2033 (6)	0.5828 (3)	0.4305 (2)	0.0373 (8)	
O2	−0.2364 (6)	0.4254 (3)	0.3645 (2)	0.0440 (9)	
O3	−0.4641 (8)	0.3955 (3)	0.2116 (3)	0.0572 (11)	
O1W	0.2550 (7)	0.3831 (3)	0.4608 (3)	0.0438 (9)	
O2W	0.2443 (8)	0.2500 (3)	0.2582 (3)	0.0632 (12)	
O3W	0.5868 (10)	0.2135 (4)	0.0401 (3)	0.0759 (13)	
O4W	0.0637 (12)	0.1102 (5)	0.0279 (6)	0.1083 (19)	
H3W	0.292 (7)	0.302 (3)	0.247 (5)	0.080*	
H7W	0.027 (14)	0.105 (5)	−0.0312 (14)	0.080*	
H1W	0.241 (5)	0.3441 (18)	0.4005 (5)	0.080*	
H5W	0.660 (10)	0.263 (3)	0.092 (3)	0.080*	
H4W	0.0823 (16)	0.237 (4)	0.253 (5)	0.080*	
H6W	0.51 (5)	0.166 (11)	0.050 (5)	0.080*	
H2W	0.407 (2)	0.3970 (11)	0.4929 (19)	0.08 (2)*	
H8W	−0.008 (8)	0.155 (3)	0.069 (3)	0.080*	

### Atomic displacement parameters (Å^2^)

**Table:**

	*U*^11^	*U*^22^	*U*^33^	*U*^12^	*U*^13^	*U*^23^
C1	0.052 (3)	0.070 (4)	0.059 (4)	0.008 (3)	0.013 (3)	0.045 (3)
C2	0.049 (3)	0.044 (3)	0.041 (3)	0.000 (2)	0.006 (2)	0.022 (2)
C3	0.056 (3)	0.042 (3)	0.039 (3)	−0.008 (3)	0.000 (2)	0.011 (2)
C4	0.059 (3)	0.047 (3)	0.059 (4)	−0.009 (3)	−0.006 (3)	0.025 (3)
C5	0.040 (3)	0.034 (2)	0.030 (3)	−0.002 (2)	0.004 (2)	0.015 (2)
C6	0.047 (3)	0.036 (3)	0.034 (3)	0.000 (2)	0.004 (2)	0.018 (2)
C7	0.038 (3)	0.030 (2)	0.030 (3)	−0.002 (2)	0.000 (2)	0.011 (2)
C8	0.036 (2)	0.034 (2)	0.028 (2)	−0.003 (2)	0.0006 (19)	0.012 (2)
C9	0.035 (2)	0.042 (3)	0.032 (3)	−0.001 (2)	0.000 (2)	0.018 (2)
C10	0.041 (3)	0.040 (3)	0.033 (3)	0.000 (2)	0.001 (2)	0.014 (2)
C11	0.031 (2)	0.037 (2)	0.029 (2)	−0.002 (2)	0.0013 (19)	0.015 (2)
C12	0.040 (3)	0.037 (3)	0.033 (3)	−0.004 (2)	−0.001 (2)	0.016 (2)
C13	0.044 (3)	0.039 (3)	0.033 (3)	0.002 (2)	−0.004 (2)	0.017 (2)
C14	0.053 (3)	0.038 (3)	0.030 (3)	−0.006 (2)	−0.001 (2)	0.015 (2)
C15	0.049 (3)	0.048 (3)	0.037 (3)	0.004 (2)	−0.007 (2)	0.024 (2)
C16	0.076 (4)	0.073 (4)	0.045 (3)	0.023 (3)	0.001 (3)	0.030 (3)
F1	0.0444 (16)	0.0568 (18)	0.0516 (19)	−0.0141 (14)	−0.0135 (13)	0.0289 (15)
Mg1	0.0354 (12)	0.0384 (12)	0.0304 (12)	−0.0011 (10)	0.0002 (9)	0.0205 (10)
N1	0.068 (3)	0.041 (3)	0.068 (3)	0.006 (2)	0.007 (3)	0.030 (2)
N2	0.053 (3)	0.039 (2)	0.033 (2)	−0.0024 (19)	0.0064 (19)	0.0177 (19)
N3	0.045 (2)	0.039 (2)	0.035 (2)	−0.0019 (19)	−0.0020 (18)	0.0209 (19)
O1	0.0381 (17)	0.0481 (19)	0.0322 (18)	0.0006 (15)	0.0023 (14)	0.0247 (15)
O2	0.0458 (19)	0.049 (2)	0.038 (2)	−0.0101 (16)	−0.0070 (15)	0.0251 (16)
O3	0.072 (3)	0.051 (2)	0.042 (2)	−0.020 (2)	−0.0172 (19)	0.0236 (18)
O1W	0.042 (2)	0.048 (2)	0.044 (2)	0.0087 (17)	0.0063 (16)	0.0205 (17)
O2W	0.056 (2)	0.059 (3)	0.075 (3)	−0.011 (2)	0.010 (2)	0.030 (2)
O3W	0.089 (3)	0.071 (3)	0.057 (3)	0.015 (3)	0.009 (2)	0.013 (2)
O4W	0.093 (4)	0.090 (4)	0.151 (6)	0.023 (3)	0.041 (4)	0.049 (4)

### Geometric parameters (Å, °)

**Table:**

C1—N1	1.458 (8)	C12—C13	1.366 (6)
C1—C2	1.498 (6)	C12—C14	1.508 (6)
C1—H1A	0.9700	C13—N3	1.335 (6)
C1—H1B	0.9700	C13—H13	0.9300
C2—N2	1.461 (6)	C14—O2	1.254 (5)
C2—H2A	0.9700	C14—O3	1.259 (6)
C2—H2B	0.9700	C15—N3	1.476 (6)
C3—N2	1.472 (6)	C15—C16	1.495 (8)
C3—C4	1.512 (7)	C15—H15A	0.9700
C3—H3A	0.9700	C15—H15B	0.9700
C3—H3B	0.9700	C16—H16A	0.9600
C4—N1	1.449 (7)	C16—H16B	0.9600
C4—H4A	0.9700	C16—H16C	0.9600
C4—H4B	0.9700	Mg1—O2^i^	2.001 (3)
C5—C6	1.389 (6)	Mg1—O2	2.001 (3)
C5—N2	1.394 (5)	Mg1—O1^i^	2.085 (3)
C5—C10	1.412 (6)	Mg1—O1	2.085 (3)
C6—C7	1.388 (6)	Mg1—O1W^i^	2.094 (3)
C6—H6	0.9300	Mg1—O1W	2.094 (3)
C7—C8	1.403 (6)	N1—H1	0.8600
C7—N3	1.408 (6)	O1W—H1W	0.820 (10)
C8—C9	1.411 (6)	O1W—H2W	0.818 (17)
C8—C11	1.443 (6)	O2W—H3W	0.82 (5)
C9—C10	1.345 (6)	O2W—H4W	0.821 (16)
C9—H9	0.9300	O3W—H5W	0.82 (4)
C10—F1	1.362 (5)	O3W—H6W	0.81 (15)
C11—O1	1.268 (5)	O4W—H8W	0.82 (4)
C11—C12	1.439 (6)	O4W—H7W	0.82 (3)
			
N1—C1—C2	110.5 (4)	N3—C13—C12	126.0 (4)
N1—C1—H1A	109.5	N3—C13—H13	117.0
C2—C1—H1A	109.5	C12—C13—H13	117.0
N1—C1—H1B	109.5	O2—C14—O3	123.8 (4)
C2—C1—H1B	109.5	O2—C14—C12	119.4 (4)
H1A—C1—H1B	108.1	O3—C14—C12	116.7 (4)
N2—C2—C1	110.1 (4)	N3—C15—C16	113.9 (4)
N2—C2—H2A	109.6	N3—C15—H15A	108.8
C1—C2—H2A	109.6	C16—C15—H15A	108.8
N2—C2—H2B	109.6	N3—C15—H15B	108.8
C1—C2—H2B	109.6	C16—C15—H15B	108.8
H2A—C2—H2B	108.1	H15A—C15—H15B	107.7
N2—C3—C4	111.2 (4)	C15—C16—H16A	109.5
N2—C3—H3A	109.4	C15—C16—H16B	109.5
C4—C3—H3A	109.4	H16A—C16—H16B	109.5
N2—C3—H3B	109.4	C15—C16—H16C	109.5
C4—C3—H3B	109.4	H16A—C16—H16C	109.5
H3A—C3—H3B	108.0	H16B—C16—H16C	109.5
N1—C4—C3	110.0 (4)	O2^i^—Mg1—O2	180.0
N1—C4—H4A	109.7	O2^i^—Mg1—O1^i^	86.80 (12)
C3—C4—H4A	109.7	O2—Mg1—O1^i^	93.20 (13)
N1—C4—H4B	109.7	O2^i^—Mg1—O1	93.20 (13)
C3—C4—H4B	109.7	O2—Mg1—O1	86.80 (12)
H4A—C4—H4B	108.2	O1^i^—Mg1—O1	180.0
C6—C5—N2	123.6 (4)	O2^i^—Mg1—O1W^i^	90.04 (15)
C6—C5—C10	115.4 (4)	O2—Mg1—O1W^i^	89.96 (15)
N2—C5—C10	121.0 (4)	O1^i^—Mg1—O1W^i^	90.47 (13)
C5—C6—C7	122.0 (4)	O1—Mg1—O1W^i^	89.53 (13)
C5—C6—H6	119.0	O2^i^—Mg1—O1W	89.96 (15)
C7—C6—H6	119.0	O2—Mg1—O1W	90.04 (14)
C6—C7—C8	121.3 (4)	O1^i^—Mg1—O1W	89.53 (13)
C6—C7—N3	120.7 (4)	O1—Mg1—O1W	90.47 (13)
C8—C7—N3	117.9 (4)	O1W^i^—Mg1—O1W	180.0
C7—C8—C9	116.7 (4)	C4—N1—C1	109.6 (4)
C7—C8—C11	122.8 (4)	C4—N1—H1	125.2
C9—C8—C11	120.5 (4)	C1—N1—H1	125.2
C10—C9—C8	120.6 (4)	C5—N2—C2	116.5 (4)
C10—C9—H9	119.7	C5—N2—C3	116.4 (4)
C8—C9—H9	119.7	C2—N2—C3	110.4 (4)
C9—C10—F1	118.5 (4)	C13—N3—C7	119.1 (4)
C9—C10—C5	123.9 (4)	C13—N3—C15	119.2 (4)
F1—C10—C5	117.6 (4)	C7—N3—C15	121.4 (4)
O1—C11—C12	123.8 (4)	C11—O1—Mg1	125.9 (3)
O1—C11—C8	120.5 (4)	C14—O2—Mg1	132.8 (3)
C12—C11—C8	115.6 (4)	H1W—O1W—H2W	115 (2)
C13—C12—C11	118.4 (4)	H3W—O2W—H4W	115 (5)
C13—C12—C14	116.7 (4)	H5W—O3W—H6W	115 (7)
C11—C12—C14	124.9 (4)	H8W—O4W—H7W	115 (6)

Symmetry codes: (i) −*x*, −*y*+1, −*z*+1.

### Hydrogen-bond geometry (Å, °)

**Table:**

*D*—H···*A*	*D*—H	H···*A*	*D*···*A*	*D*—H···*A*
N1—H1···O2W^ii^	0.86	2.20	2.787 (7)	126
O1W—H1W···O2W	0.82 (1)	1.97 (1)	2.786 (6)	175 (3)
O1W—H2W···O1^iii^	0.82 (2)	2.09 (2)	2.901 (5)	175 (2)
O2W—H3W···O3^iv^	0.82 (5)	1.98 (5)	2.749 (6)	156 (4)
O2W—H4W···N1^v^	0.82 (2)	2.36 (4)	3.121 (7)	154 (5)
O3W—H5W···O3^iv^	0.82 (4)	2.18 (4)	2.835 (6)	137 (5)
O3W—H6W···O4W	0.81 (15)	2.3 (2)	2.890 (8)	131 (19)
O4W—H8W···O3W^vi^	0.82 (4)	2.31 (4)	2.888 (8)	128 (4)

Symmetry codes: (ii) *x*, *y*+1, *z*; (iii) −*x*+1, −*y*+1, −*z*+1; (iv) *x*+1, *y*, *z*; (v) *x*−1, *y*−1, *z*; (vi) *x*−1, *y*, *z*.
